# Dengue Fever and Severe Dengue in Barbados, 2008–2016

**DOI:** 10.3390/tropicalmed5020068

**Published:** 2020-05-02

**Authors:** Kirk Osmond Douglas, Sudip Kumar Dutta, Byron Martina, Fatih Anfasa, T. Alafia Samuels, Marquita Gittens-St. Hilaire

**Affiliations:** 1 Faculty of Medical Sciences, University of the West Indies, Cave Hill, St. Michael BB11000, Barbados; marquita.gittens@cavehill.uwi.edu; 2 Artemis One Health Research Foundation, Molengraaffsingel 10, 2629 JD Delft, The Netherlands; s.dutta@artemisonehealth.com (S.K.D.); b.martina@artemisonehealth.com (B.M.); 3 Department of Viroscience, Postgraduate School Molecular Medicine, Erasmus University Medical Center, Wytemaweg 80, 3015 CN Rotterdam, The Netherlands; fatih.anfasa@gmail.com; 4 Department of Internal Medicine, Faculty of Medicine, Universitas Indonesia, Diponegoro 71, Central Jakarta 10430, Indonesia; 5 Epidemiology Research Unit, Caribbean Institute for Health Research (CAIHR), University of the West Indies, Mona, Kingston 7, Jamaica; alafia.samuels@cavehill.uwi.edu; 6 Best–dos Santos Public Health Laboratory, Enmore Complex, Martindales Road, St. Michael BB11155, Barbados

**Keywords:** dengue, severe dengue, genetic sequencing, DENV, epidemiology, arbovirus, public health, epidemics, Barbados, Caribbean

## Abstract

Analysis of the temporal, seasonal and demographic distribution of dengue virus (DENV) infections in Barbados was conducted using national surveillance data from a total of 3994 confirmed dengue cases. Diagnosis was confirmed either by DENV–specific real time reverse transcriptase polymerase chain reaction (rRT–PCR), or non–structural protein 1 (NS1) antigen or enzyme linked immunosorbent assay (ELISA) tests; a case fatality rate of 0.4% (10/3994) was observed. The dengue fever (DF) prevalence varied from 27.5 to 453.9 cases per 100,000 population among febrile patients who sought medical attention annually. DF cases occurred throughout the year with low level of transmission observed during the dry season (December to June), then increased transmission during rainy season (July to November) peaking in October. Three major dengue epidemics occurred in Barbados during 2010, 2013 and possibly 2016 with an emerging three–year interval. DF prevalence among febrile patients who sought medical attention overall was highest among the 10–19 years old age group. The highest DF hospitalisation prevalence was observed in 2013. Multiple serotypes circulated during the study period and Dengue virus serotype 2 (DENV–2) was the most prevalent serotype during 2010, whilst DENV–1 was the most prevalent serotype in 2013. Two DENV–1 strains from the 2013 DENV epidemic were genetically more closely related to South East Asian strains, than Caribbean or South American strains, and represent the first ever sequencing of DENV strains in Barbados. However, the small sample size (*n* = 2) limits any meaningful conclusions. DF prevalence was not significantly different between females and males. Public health planning should consider DENV inter–epidemic periodicity, the current COVID–19 pandemic and similar clinical symptomology between DF and COVID–19. The implementation of routine sequencing of DENV strains to obtain critical data can aid in battling DENV epidemics in Barbados.

## 1. Introduction

Dengue fever (DF) is the most prevalent human arboviral disease in tropical and subtropical regions worldwide. Dengue virus (DENV) is a global health threat that is primarily acquired through the bites of infected mosquitoes and responsible for over 100 million infections and 20,000 deaths annually [1,2].

Current dengue epidemiology research aids in vaccine planning initiatives as it describes the transmission dynamics within a population and the possible risk factors for DENV infections. In the Latin America and Caribbean, such epidemiological data is varied in approach which can lead to inconsistencies on the impact of age on disease burden and severity [3]. Dengue has been endemic in Barbados for over 30 years with the circulation of all four DENV serotypes [4,5,6,7,8,9,10,11,12]. Barbados has two annual seasons namely a dry and wet season. The dry season occurs from December–May and the wet/rainy season from June to November. DENV infection is transmitted year–round in Barbados and dengue epidemics have occurred in 1995, 1997, 2001, and 2007 [6,7,9]. Notably, dengue haemorrhagic fever (DHF) in Barbados and the Caribbean does not appear to be as prevalent as in other Asian countries where dengue is more severe. Current systematic population data on dengue epidemiology in Barbados within the last 10 years has been absent. A recent dengue epidemiology study involved children up to 16 years old (2000–2009) but lacked detailed data on the entire population [8].

We present epidemiological data across Barbados from a centralised laboratory where all suspected and confirmed dengue cases in the island are tested. We also performed virus sequencing on the envelope (E) gene of two DENV strains that were obtained from two real–time reverse transcriptase polymerase chain reaction– (rRT–PCR) positive out of 33 patient sera.

## 2. Materials and Methods

### 2.1. Ethical Approval

The study (IRB No. 181110–A) was approved by Institutional Review Board (IRB) on Ethics in Research on Human Subjects at The University of the West Indies (UWI), Cave Hill, St. Michael, Barbados combined with the Ministry of Health on 11 July 2013 and the Ethics Committee at the Queen Elizabeth Hospital (QEH), Martindale’s Road, St. Michael, Barbados on 19 August 2013 prior to the start of data collection and analyses.

### 2.2. Sample Collection

All suspected febrile patients who seek medical attention on the island are referred to the central public health laboratory based on the similarity of clinical symptoms of DENV, chikungunya virus (CHIKV), Zika virus (ZIKV), *Leptospira* and hantavirus infections including fever, malaise, myalgia, arthralgia, rash, retro–orbital pain, abdominal pain, nausea and vomiting. Random sampling of patients from this database then permits a good representation of the entire population in Barbados with febrile illness who sought medical attention. Dengue is a reportable disease in Barbados using a functional passive surveillance system. The amalgamated Barbados public health laboratory is the sole laboratory where all suspected dengue/leptospirosis febrile patients are tested for confirmation of DENV, hantavirus, CHIKV, ZIKV and *Leptospira* infections.

All dengue cases, including probable and confirmed cases were diagnosed per the case definitions issued by Barbados Ministry of Health. A DF and DHF case were defined as per the 1997 WHO dengue guidelines for 2008 and a DF and severe dengue (SD) case were defined as per the 2009 WHO dengue guidelines for 2009–2016. Dengue cases were registered using surveillance forms issued by the Barbados Ministry of Health. These data were from suspected febrile patients tested for several infections including DENV, *Leptospira*, CHIKV, ZIKV and hantavirus. A dengue epidemic was defined by a significant difference in DF prevalence between the preceding year and the epidemic year where the DF prevalence is significantly higher in the epidemic year compared to the preceding year.

**Figure 1:**
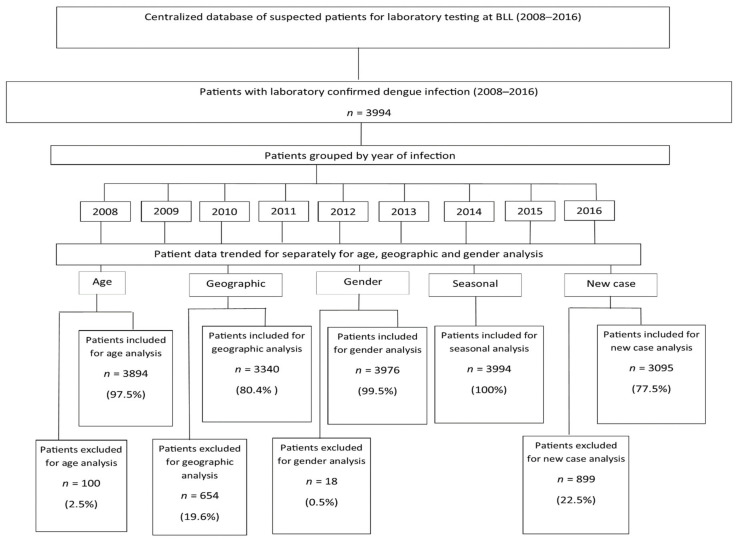
Sampling and analysis of dengue fever (DF) patients, Barbados, 2008–2016.

Using a centralised patient database at the laboratory, a list of 3994 laboratory confirmed dengue cases were identified by DENV–specific rRT–PCR, immunoglobulin M (IgM) and immunoglobulin G (IgG) enzyme linked immunosorbent assay (ELISA) and non-specific protein 1 (NS1) antigen testing in Barbados during 2008 to 2016. This list of patients was analysed and pertinent epidemiological characteristics of infection including age, new dengue case, gender and geographical location during this period were reported. The list of patients was exported from Microsoft Access as a Microsoft Excel file for analysis. The patients were first grouped by year of dengue infection for the period of study 2008–2016 (Figure 1). Using Microsoft Excel, each of the groups were sorted by age and the list of total patients was reduced to 3894 out of the original 3994 patients (97.5%) as some of patients’ ages were not recorded. Each year group was sorted by age and the tally of each age group recorded. Age standardisation was done using World Health Organization (WHO) standard [13].

For analysis by gender, all 3994 laboratory confirmed dengue patients were used (100%) as all patients had their gender recorded. Each group was sorted by gender and the tally of patients for each gender was recorded. For analysis by geographic location, only 80.4% (3340/3994) were used as a parish address was missing for 19.6% of dengue cases on the list generated. From these, 3340 dengue patients each group was sorted by parish and the tally of patients for each parish was recorded. For analysis of new dengue cases, 3095 laboratory confirmed dengue patients were used (77.5%) with both IgM and IgG results. These new cases were sorted by age and gender and the tallies of patients were recorded.

### 2.3. Dengue Diagnosis

Laboratory confirmation of dengue cases were conducted via either detection of DENV–specific IgM and IgG in patients’ serum with dengue IgM and IgG antibody–capture ELISA kits (samples within 5–15 days of illness), or NS1 antigen or rRT–PCR (samples within the first five days of illness). IgG and IgM antibody–capture ELISAs were performed per manufacturer’s instructions (Focus Diagnostics, Cypress, CA90630 USA) [14,15]. Platelia™ Dengue NS1 Ag–ELISA (Biorad Laboratories, Marnes–La–Coquette, France) was used for the NS1 antigen detection [16]. The patient sera were tested using rRT–PCR to serotype the infecting DENV strain(s) as per the public health laboratory’s dengue RT–PCR serotyping procedure [17]. For each testing assay, the relevant kit and in–house positive and negative controls were used for quality control. A primary/new dengue case was defined by either the presence of DENV–specific viral RNA (vRNA), NS1 antigen or DENV–specific IgM along with the absence of DENV–specific IgG.

### 2.4. Virus Sequencing—Next Generation Sequencing (NGS)

Primers were designed for the E gene that allowed five fragments of sizes between up to 500 nucleotides, with about 50 nucleotides of overlap to be generated. Real time reverse transcriptase polymerase chain reaction (rRT-PCR) was conducted using random primers (Invitrogen) and Superscript III (Invitrogen), and deoxyribonucleotide (DNA) amplification was performed using the specific primers and PFU polymerase (Invitrogen). Fragments were gel–purified using QIAquick gel extraction kit (Qiagen, Venlo, The Netherlands) and were organized in libraries of equal concentrations. Libraries were created for each virus without DNA fragmentation (GS FLX Titanium Rapid Library Preparation, Roche), emPCR (Amplification Method Lib–L) and GS junior sequencing runs were performed according to instructions of the manufacturer (Roche). Amplicons were sequenced in a blinded fashion using 454 technology. Reads from the GS–FLX sequencing data were sorted by bar code and aligned to reference sequences using CLC Genomics software 4.6.1. Using the alignment, a single nucleotide polymorphism (SNP) table was made with a minimum coverage of 10 reads and a minimum variant frequency of 1.0%. Raw nucleotide sequences were filtered, aligned, trimmed and translated using pre–specified criteria applied uniformly so that all the protein E sequences used in the analyses spanned the exo–domain and the transmembrane region.

### 2.5. Phylogenetic Analysis

The phylogenetic analysis was performed using MEGA version 6, considering sequences dengue virus envelope (DENV–E) gene obtained in the present study (viz. DS18 and DS29) with global reference sequences of different dengue serotype (DENV–1, –2, –3 and –4) and DENV–1 sequences from different geographic regions, available in the GenBank database. The nucleotide sequences were aligned using MUSCLE v.3.6. and the phylogenetic tree was generated considering complete envelop gene (E) of DENV, using the maximal likelihood method (ML) based on Tamura–Nei (TN93) + gamma model. Reliability of the analysis was evaluated by a bootstrap test with 1000 replications.

### 2.6. Data Analysis

Data analysis was done using Microsoft Excel (Microsoft, Redmond, WA, USA) and SPSS 16.0 software (SPSS Inc., Chicago, IL, USA) for statistical analysis. Confidence intervals (95%) were calculated for prevalence, case fatality risk/rate (CFR) and hospitalisation prevalence using Barbados 2010 census data and Barbados population data from 2014. CFR is the number of persons who died ÷ number of persons infected. A probability (*p*) of ≤ 0.05 was considered statistically significant.

## 3. Results

### 3.1. Laboratory Testing and Clinical Symptoms

The largest number of diagnostic tests were performed in 2013 (Table 1). The most common clinical symptoms observed among DENV–infected patients in Barbados were fever, headache, joint pain, retro–orbital pain, muscle pain and gastrointestinal symptoms (Table 2). More severe symptoms such as bleeding, jaundice, thrombocytopenia and hepatomegaly (usually <12%) were not observed in a high proportion of DENV cases (Table 2).

### 3.2. Epidemiological Patterns of Dengue in Barbados

**Figure 2:**
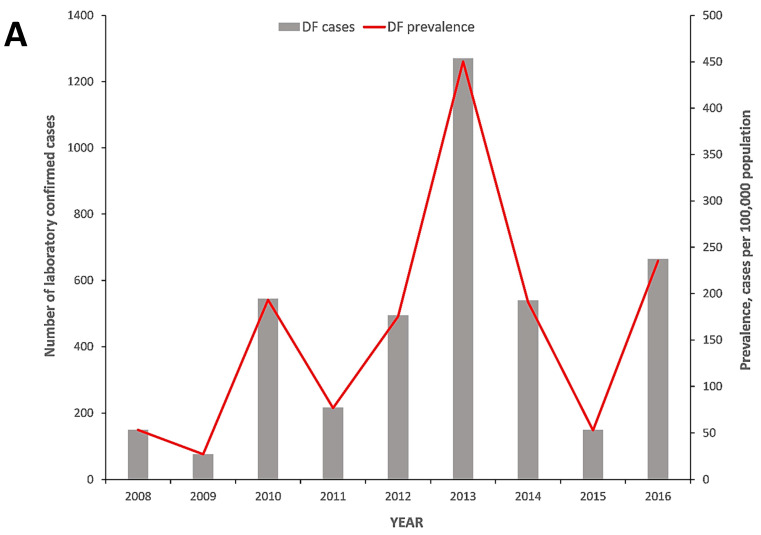

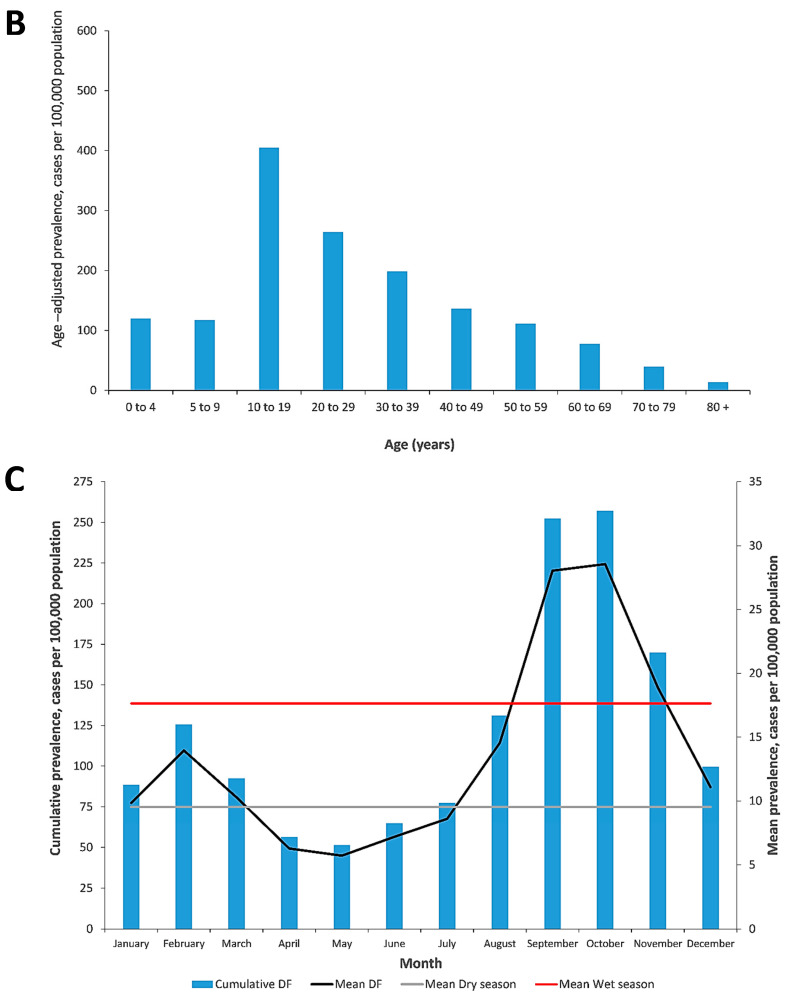
(**A**) Number of dengue fever (DF) cases and prevalence among patients seeking medical attention in Barbados, 2008–2016; (**B**) DF prevalence and age groups among patients seeking medical attention in Barbados, 2008–2016; (**C**) monthly cumulative DF, mean DF, mean dry season and mean wet season prevalence among patients seeking medical attention in Barbados, 2008–2016.

During 2008–2016, a total of 3994 laboratory confirmed dengue cases were reported in Barbados. The DF prevalence varied by year from 27.4 to 453.4 cases per 100,000 population annually (Figure 2A). The emergence of a three–year inter–epidemic interval with DF prevalence peaks in 2010, 2013 and 2016 were observed (Figure 2A). Statistical analysis showed significant differences in annual DF prevalence between the year preceding and the epidemic year for 2009 vs. 2010, (27.4 (95% CI, 21.2–33.5) and 194.7 (95% CI, 178.3–211.1) cases per 100,000 population, respectively), 2012 vs. 2013, (176.7 (95% CI, 161.0–192.4) and 453.9 (95% CI, 428.8–478.9) cases per 100,000 population, respectively) and 2015 vs. 2016 (53.3 (95% CI, 44.7–61.9) and 237.2 (95% CI, 219.1–255.3) cases per 100,000 population, respectively) (Figure 2A). Thus, major dengue epidemics occurred in 2010, 2013 and 2016; the 2013 epidemic started in late 2012, peaked in 2013 and spilled over into 2014 (Figure 2A; data not shown). The highest DF prevalence was observed during 2013 and was significantly higher than all the years in the study period (Figure 2A).

The highest age adjusted DF prevalence was observed in patients 10–19 years of age with 404.7 (95% CI, 378.2–431.4) cases per 100,000 population, which was significantly higher than all other age groups, and the next highest was the 20–29 age group 264.3 (95% CI, 243.4–285.4) cases per 100,000 population (Figure 2B).

The mean monthly DF prevalence in Barbados differed significantly from the rainy season (June to November), with 17.6 (95% CI, 16.0–19.3) cases per 100,000 population, to the dry season (December to May), with 9.5 (95% CI, 7.9–11.1) cases per 100,000 population (Figure 2C). During the study period, the cumulative DF prevalence increased from June, peaking in October, with 257.8 (95% CI, 238.2–275.9) cases per 100,000 population, then decreasing to lower levels in December (Figure 2C). Consistently low cumulative DF prevalence occurred from January to May (Figure 2C).

All four DENV serotypes were observed during the study period 2008–2016, with DENV–2 being the predominant DENV serotype observed in 2010 and 2011, whilst DENV–1 became the predominant DENV serotype in 2012 and 2013 (Table 1). DENV serotyping was absent for 2008 and 2014–2016 (Table 1). The predominant serotypes during 2010 and 2013 epidemics were DENV–2 and DENV–1, respectively, and may explain the fewer SD cases observed in 2013 than in 2010 (Table 1). DENV–3 serotype was the most dominant serotype in 2007; therefore, is it not unusual that the DENV epidemics observed in 2010 and 2013 were caused predominantly by DENV–2 and DENV–1, respectively.

**Figure 3:**
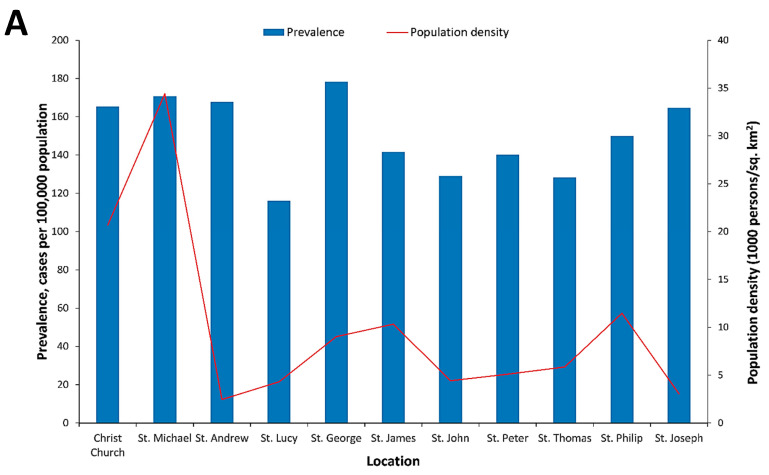

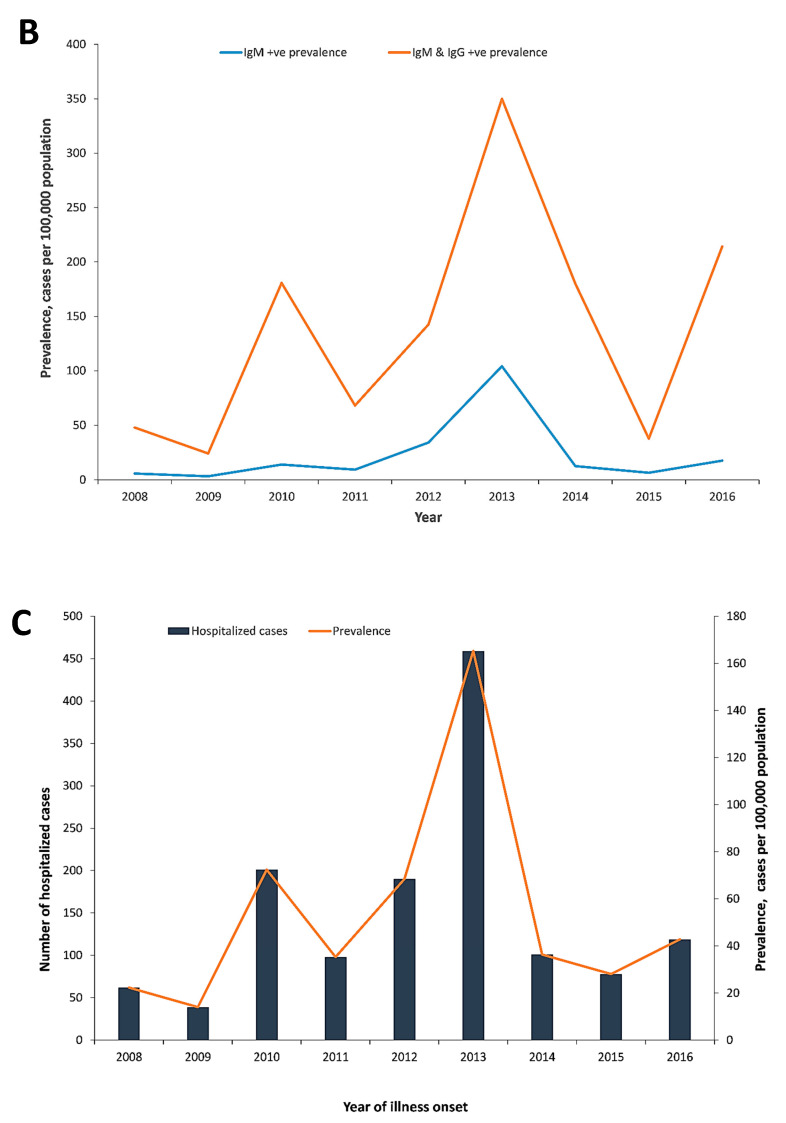
(**A**) Dengue fever (DF) prevalence by geographic location among patients seeking medical attention in Barbados, 2008–2016; (**B**) primary (IgM positive only) and secondary (IgM and IgG positive) DF prevalence for patients seeking medical attention in Barbados, 2008–2016; (**C**) number of hospitalized DF cases and hospitalized DF prevalence among patients seeking medical attention in Barbados, 2008–2016.

During the 2010 and 2013 dengue epidemics, the geographic distribution of dengue infections in Barbados was different. No significant difference was observed in crude DF prevalence among different parishes (Figure 3A). No significant difference was observed for the mean crude DF prevalence between males and females: A total of 153.6 (95% CI, 146.3–164.4) vs. 164.7 (95% CI, 158.2–168.5) cases per 100,000 population respectively; a ratio of 1:1.

A total of 576 primary dengue cases were observed in the period 2008–2016. The highest primary dengue prevalence were observed in 2013, with 104.0 (95% CI, 92.0–116.0) cases per 100,000 population, and 2012, with 34.2 (95% CI, 27.3–41.1) cases per 100,000 population, which were significantly higher than other years (Figure 3B). Primary DENV infection accounted for 576 confirmed patients (14.4%), and at least 2382 (59.6%) secondary DENV infections occurred during 2008 to 2016.

### 3.3. Severe Dengue in Barbados

**Figure 4:**
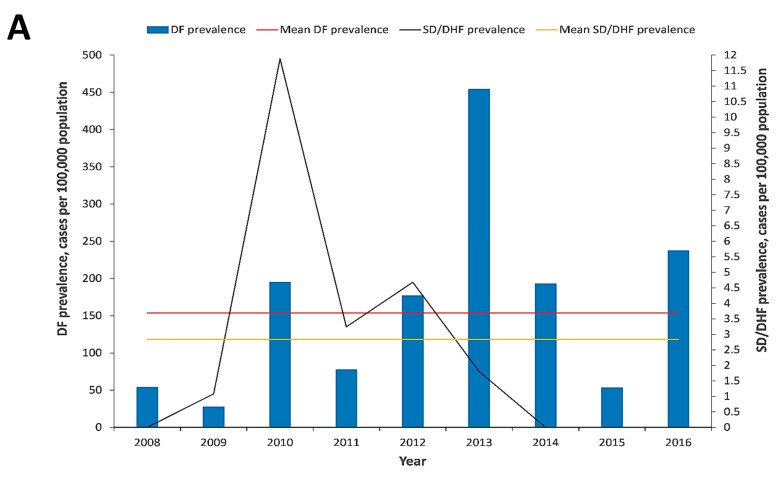

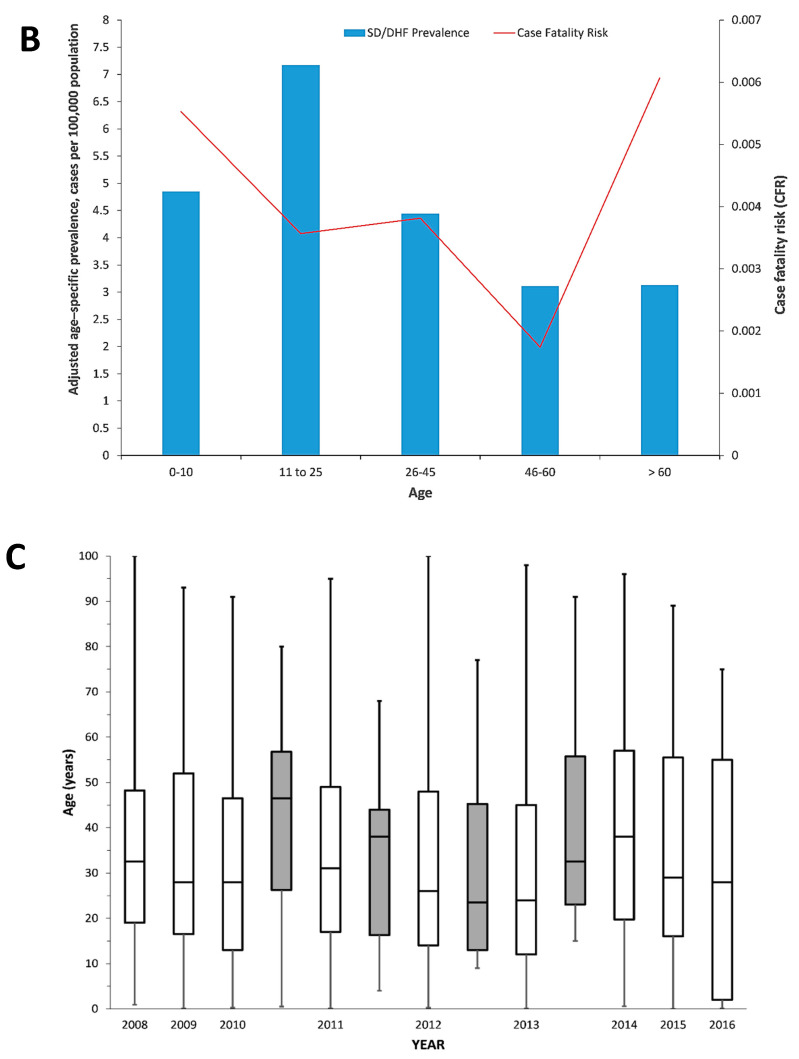
(**A**) Dengue fever (DF), mean DF, severe dengue (SD)/dengue haemorrhagic fever (DHF) and mean SD/DHF prevalence among patients seeking medical attention in Barbados, 2008–2016; (**B**) SD/DHF prevalence and case fatality risk (CFR) by age groups among patients seeking medical attention in Barbados, 2008–2016; (**C**) DF and SD/DHF age distribution among patients seeking medical attention in Barbados, 2008–2016. Boxes encompass 25th and 75th percentiles. Black lines within the boxes represent medians. Error bars represent the minimum and maximum ranges.

DENV infection in 1356 out of 3994 (34.0%) patients resulted in hospitalizations, and the crude prevalence of hospitalized DF patients ranged from 14.2 (95% CI, 8.2–19.8) cases per 100,000 population to 165.2 (95% CI, 150.1–180.3) cases per 100,000 population between 2008 and 2016 (Figure 3C). During 2013, the crude prevalence of hospitalized DF patients, with 165.2 (95% CI, 150.1–180.3) cases per 100,000 population, was significantly higher than every year during the study and was followed by the crude hospitalized dengue prevalence in 2010, with 72.4 (95% CI, 62.3–82.4) cases per 100,000 population (Figure 3C). SD/DHF prevalence varied from 0 to 11.9 (95% CI, 7.8–15.9) cases per 100,000 population (Figure 4A). The mean SD/DHF prevalence observed during the study period was 2.5 (95% CI, 1.2–3.8) cases per 100,000 population. A significantly higher SD/DHF prevalence was observed during 2010, 11.9 (95% CI, 7.8–15.9) cases per 100,000 population than in every other year in the study, even though a higher DENV prevalence was observed in 2013 than in 2010 (Figure 2A and Figure 4A). The 2010 dengue epidemic was more severe than 2013 epidemic as the SD/DHF prevalence in 2010, with 11.9 (95% CI, 7.8–15.9) cases per 100,000 population, was significantly higher than the SD/DHF prevalence in 2013, with 1.8 (95% CI, 0.2–3.4) cases per 100,000 population (Figure 4A).

The CFR among SD/DHF cases varied by age and ranged from 0.0017 to 0.0061, and was highest among the elderly, persons 60 years or older and the young (0.075 per 100 DF cases), even though the age adjusted prevalence was higher in the 11–25 years old age group (Figure 4B). An overall CFR of (10/3994) 0.4% was observed over the study period.

Median age of SD/DHF patients varied during 2008–2016, with the highest median age observed in 2010 (Figure 4C). Most SD/DHF cases in 2012 were young (<30 years old), while in 2010 most DHF cases were older persons (>30 years old; Figure 4C). The age distribution of the DF patients ranged from one to 100 years (median 29.6 years) and the median age varied over time (Figure 4C). The median age of SD/DHF cases was 40 years old (interquartile age range of 23–56 years old) and the median age varied between 2010 to 2013 (Figure 4C). SD cases were observed in 2009–2013 and an overall mean age of 35.8 years old was observed over the study period (Figure 4C).

### 3.4. DENV Sequencing in Dengue Patient Sera

**Figure 5:**
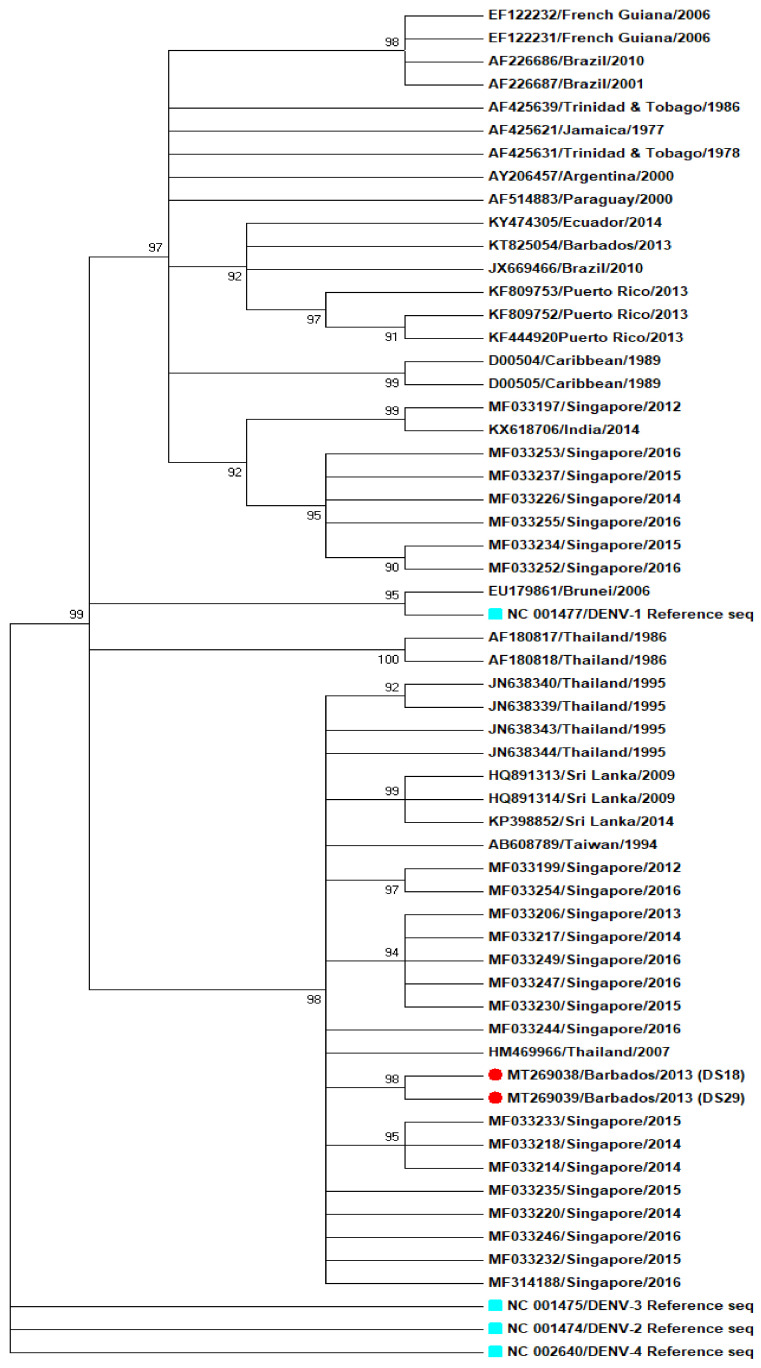
Phylogenetic analysis of dengue virus type 1 (DENV–1), based on complete envelop gene (E). The tree was constructed using maximum likelihood (ML) method, with 1000 boot strap resampling. The boot strap values were mention adjacent to the branch. Patient sample DS18 (MT269038) and DS29 (MT269039) were marked with red dots, different serotypes of dengue NCBI reference strains were marked with cyan dots.

Two out of thirty–three (2/33) DENV specific rRT–PCR positive sera samples yielded E gene sequences using NGS methods (Figure 5). The viral loads on the remaining 31 samples were too low to permit sequencing using NGS. Two samples were positive in the rRT–PCR, patients DS18 and DS29, with cycle threshold (Ct) values of, respectively, 28.5 and 30. Both sequences, GenBank accession numbers MT269038 (from DS18) and MT269039 (from DS29), grouped closer to DENV–1 strains of South–East (SE) Asian origin than to DENV–1 strains from Caribbean or South American origin; however, the small sample size (*n* = 2) limits the drawing of any meaningful conclusions (Figure 5).

## 4. Discussion

Epidemiological data are important in vaccine planning and are critical in measuring vaccine efficacy and disease burden [18]. The mean dengue prevalence observed in this study was comparable with previous studies in Barbados (163.0 vs. 162.5 cases per 100,000 population) and more than double that of other countries within the Caribbean and Latin America (163.0 vs. 72.1 cases per 100,000; *P* < 0.01) [19]. This prevalence remains one of the highest in Caribbean along with Trinidad, Martinique, Guadeloupe, French Guiana and Puerto Rico [19]. However other Caribbean territories have significantly lower prevalence, as low as 140 times lower [19]. These disparities in DF prevalence may be due to differences in the extensiveness of the DENV laboratory diagnostic testing and laboratory surveillance systems in each English–speaking Caribbean country. The more extensive the reporting, the more accurate the prevalence. Other factors that contribute are reduced public health awareness, population density, infecting DENV strain, urban planning activities, topography, water storage practices, change of lifestyle, apathy and heightened medical seeking behaviour [20,21,22,23]. Variation in the number of laboratory tests conducted can also influence this was well as more tests conducted will results in more cases detected. A cross–sectional serological survey within the community may be more representative and accurate as it would be a random sampling of the population without the bias of patients seeking medical attention which can result in higher prevalence.

A period between dengue epidemics is often observed though the periodic length may vary but usually is 3 to 5 years in length [12,24,25,26]. A cyclic pattern of dengue epidemics every 3 years has been observed in Barbados since 2007 including 2007, 2010, 2013 and possibly 2016 as evidenced by peak prevalence in the period of study [9]. It is noted that ZIKV was first detected in Barbados during 2016 and the introduction of ZIKV likely increased the number of persons with DF–like symptoms having flavivirus IgM positive serological results. Only ZIKV molecular testing can conclusively determine the nature of the clinical infection(s). If interepidemic period of 3–5 years is consistent the next DENV epidemic in Barbados is likely due in 2020 or 2021 and the possibility of the concurrent occurrence of severe acute respiratory syndrome coronavirus 2 (SARS CoV–2) pandemic and a DENV epidemic exists. Reports of suspected DENV infections later being confirmed as SARS–CoV–2 infections and the similarity with clinical symptoms could lead to complication of SARS–CoV–2 pandemic response [27,28,29,30].

Median age is a predictor of the change in force of infection and mean force of infection [18]. Force of infection is defined as the rate at which susceptible individuals acquire an infectious disease and it is dependent on several factors including disease prevalence, rate of contact of individuals (communicable diseases) and infectiousness of individuals [18]. As the median age increases, the force of infection decreases. The age groups most affected by dengue and DHF/SD can differ in different geographical locations including SE Asia, Europe and the Americas based partly on differences in the relative force of infection [31,32,33]. Dengue endemic areas have a higher force of infection due to the greater exposure risk to DENV infection by vectors and the higher number of new DENV infections. The high DF prevalence observed in patients 10–19 years is consistent with prevalence data obtained from other DF endemic locations including Laos [34].

Previous studies have illustrated that gentrification, a shift of the age distribution of human population toward older ages, increases the risk of severe dengue prevalence [35,36]. In endemic countries, such as Barbados, the greater proportion of DENV infections were secondary occurring in persons <20 years. Conversely, for non–endemic countries such as mainland USA, the majority of DENV infections are primary in nature and occur in older persons 21–60 years old [37]. The implication of a higher proportion of the population with secondary DENV infection is a higher risk of developing DHF/SD [38]. The lower the median age of secondary DENV infection the higher is the risk of developing DHF/SD for younger persons since risk of developing SD/DHF increases with secondary infection.

### 4.1. Seasonal Factors

Climatic factors can enhance vector population growth and increase the risk of DENV vectors biting susceptible humans [39,40,41]. Dengue transmission is often seasonal and driven by many climatic factors including temperature, short and long term rainfall and relative humidity influencing mosquito vector growth and density [42,43,44,45]. The significant difference between DF prevalence during the rainy and dry seasons in Barbados highlights rainfall as a key factor in DENV transmission as it permits the proliferation of dengue vectors and increased risk of bites by DENV infected vectors as observed previously [46,47,48,49].

Primarily a higher DF prevalence is observed in urban areas versus rural areas as the population density is higher in urban areas resulting in more susceptible human hosts [50] however in Barbados, no significant difference in DF prevalence was observed among parishes during the study period. Population density alone does not influence dengue transmission as other factors such as degree of vegetation cover and availability of water supply could also influence [51,52,53,54].

### 4.2. Dengue and Gender

Sex bias does occur in infectious disease epidemiology including dengue infections [55]. Among patients tested positive for DENV infection, males were as likely as females to be infected with DENV during the study period in Barbados. Higher prevalence of DENV infection were observed in males from studies in Asia while reports from the Americas showed either females are more often infected or the proportion is nearly equal [55,56,57,58,59]. In Asia this disparity was linked to more males in the labour force and working outdoors [60]. Unlike in Singapore where there are more working males than females, the skilled labour force in Barbados has a similar number of males and females, 30,454 males vs. 29,872 females, (2010 Barbados census data) [60].

### 4.3. Dengue Disease Severity

In the Americas, the severe forms of dengue are not as common as in Southeast (SE) Asia as DHF or DSS attack rates are 18–fold greater in SE Asia than in the Americas [61]. In comparison the mean SD/DHF prevalence in Barbados is lower than Indonesia (30–85 cases per 100,000 population) and several other to SE Asian countries [62,63]. Mean SD/DHF prevalence in this study was comparatively higher to previous studies in Barbados, and other Caribbean countries excluding Trinidad, Turks and Caicos, Suriname, Bermuda, Montserrat and Cayman Islands [19]. One possible explanation is the improvement of DENV surveillance in Barbados. However, there could be other possibilities such as viral strain differences, public health awareness, basis of surveillance (i.e., population based), population age structure, and effectiveness of vector control programs. An example is the use of only rapid test kits with low sensitivity to diagnose dengue. Moreover, only samples of severe cases that are send to CARPHA for more sensitive diagnostic testing.

Among SD/DHF cases in Barbados, the highest prevalence was observed among persons 11–25 years of age which is slightly higher than other countries including Brazil, Sri Lanka and Thailand where SD/DHF is mostly observed in persons aged 15 or younger [64,65]. Younger members of the population are more susceptible to dengue infection as they lack substantial immunity however, but this immunity increases with age as observed in the decline in DF prevalence as age increased. Even though dengue prevalence has increased in Barbados as in other areas [66,67,68], attenuation of the severity has occurred, resulting in a shift in the age distribution of DHF toward older age groups [66,69,70].

The CFR in Barbados is relatively lower than dengue CFRs in most dengue endemic countries except for Singapore during 2004 and Vietnam during 2010 [62,63]. This lower CFR maybe due to several factors including easier access to health care, effective mosquito control, water storage practices, host population genetics, vector biology, geophysical factors and other socioeconomic differences [49,51,64]. Fatal dengue cases did occur during the study period reviewed but these were likely underestimated as the reliance on passive case reporting and/or review of death certificates can fail to identify fatal cases due to misdiagnosis [71,72,73,74,75,76]. No gender bias exists in Barbados for DHF/SD as in other countries since males (*n* = 35) were as likely as females (*n* = 38) to be infected with DENV and develop DHF or SD during the study period [55].

All 4 DENV serotypes were in circulation during 2010 epidemic and this likely increased the risk of developing SD/DHF [12,77,78]. The infecting DENV strain influences the severity of dengue disease and the infecting strains in 2010 likely differed from those in 2013 [79]. Sequence data from DENVs in circulation especially during epidemics can provide useful information regarding strain ‘fitness’ and the ability to cause severe disease thus should be included as part of routine dengue surveillance and public health plans [80,81,82].

### 4.4. Sequencing of DENV Envelope (E) Gene Region

DENV–1 serotype has been present in the Caribbean since 1970s, being first reported in Jamaica and was reported as the first DENV serotype to enter Brazil from the Caribbean [83,84]. One study revealed that DENV–1 was first present in Grenada in the Lesser Antilles, then rapidly spread to South and Central America and then to the Greater Antilles [83,84]. One phylogenetic study of DENV–1 envelope genes revealed the role of East Asian countries as possible sources of dengue epidemics and the close relatedness of at least one Caribbean DENV–1 virus to East Asian DENV–1 viruses [85]. The E gene sequence of two RT–PCR positive patient sera samples yielded E gene sequences closer in relation to DENV–1 isolates of Asian origin, Thailand and Singapore than those from the Caribbean and South America. Sequencing of DENVs from 2010 outbreak would have been of great interest to observe their genetic relationship with other sequenced DENVs. However, no deeper insights into DENVs strain can be since the sample number of sequenced strains (*n* = 2) is too small. More sequence data from different time points would be important to perform a thorough analysis.

### 4.5. Study Limitations

Our study has several limitations including selection bias due to the selection and testing of patients based on clinical suspicion and is biased due to surveillance, incomplete referral and dengue diagnostic testing, the potential to miss mild cases and non–symptomatic dengue cases and persistence of DENV–specific IgM [86]. There are however several strengths of this study due to the key observations obtained including (a) first sequencing of DENVs from Barbados which bear closer relation to Asian strains than Caribbean or South American ones, (b) a DENV epidemic periodicity of 3–5 years and the possible complications for public health officials with current coronavirus disease 2019 (COVID–19) pandemic planning, (c) lack of gender bias of DENV infection and d) generally a low DENV CFR.

### 4.6. Future Research

Future research efforts should include analysis of sociodemographic and socioeconomic determinants of DENV infection, DENV and arboviral research in mosquito vectors to identify DENV strains in circulation, DENV, ZIKV and CHIKV diagnostic testing of donated blood for blood banking, DENV seroprevalence studies for any future DENV vaccination efforts and genetic characterisation of DENV strains to assess the evolution of DENV in Barbados.

## Figures and Tables

**Table 1:** Epidemiological and laboratory testing features of dengue virus (DENV) infections in Barbados, 2008–2016.

Year	No. of Cases	Males	Females	DENV Serotypes	No. of SD/DHF Cases	No. of Deaths	Diagnostic Testing		
							ELISA IgM	NS1	rRT–PCR
2008	147	70	77	n/a	0	0	147	–	–
2009	76	35	40	3	3	0	76	–	–
2010	542	265	275	1,2 *,3,4	33	3	491	55	5
2011	217	103	114	1,2 *,4	9	5	206	16	11
2012	464	218	245	1 *,2,4	13	2	428	85	78
2013	1260	594	665	1 *,2,4	5	0	947	375	143
2014	523	248	274	n/a	0	0	521	14	–
2015	123	72	52	n/a	0	0	124	4	–
2016	642	406	227	n/a	0	0	642	3	23
TOTAL	3994	2011	1969		63	10	3582	548	237

*—Predominant DENV subtype; n/a—not available. Note: All patients with DENV–specific enzyme linked immunosorbent assay (ELISA) immunoglobulin G (IgG) negative ELISA results in combination with either DENV–positive ELISA immunoglobulin M (IgM), non–specific protein 1 (NS1) or real–time reverse transcriptase polymerase chain reaction (RT–PCR) positive results were considered primary/new dengue cases.

**Table 2:** Frequency of clinical symptoms in dengue fever (DF) patients in Barbados (2008–2016).

Symptoms	No. of Patients	Frequency (%)
Fever	3702	92.7
Headache	2471	61.9
Joint pain	2103	52.7
Eye pain	1655	41.4
Muscle pain	1533	38.4
Gastrointestinal related symptoms *	1163	29.1
Rash	981	24.6
Anorexia	846	21.2
Bleeding	467	11.7
Respiratory symptoms	308	7.7
Thrombocytopenia	256	6.4
Jaundice	179	4.5
Lethargy	89	2.2
Cough	82	2.1
Hepatomegaly	52	1.3

* Gastrointestinal–related symptoms include vomiting, abdominal pain and diarrhoea.
